# Cortical Complexity in Anorexia Nervosa: A Fractal Dimension Analysis

**DOI:** 10.3390/jcm9030833

**Published:** 2020-03-19

**Authors:** Enrico Collantoni, Christopher R. Madan, Paolo Meneguzzo, Iolanna Chiappini, Elena Tenconi, Renzo Manara, Angela Favaro

**Affiliations:** 1 Department of Neurosciences, University of Padua, Via Giustiniani, 2-35128 Padova, Italy; meneguzzo.p@gmail.com (P.M.); iolanna.chiappini@gmail.com (I.C.); elena.tenconi@unipd.it (E.T.); renzo.manara@unipd.it (R.M.); angela.favaro@unipd.it (A.F.); 2 School of Psychology, University of Nottingham, Nottingham NG7 2QL, UK; Christopher.Madan@nottingham.ac.uk; 3 Padua Neuroscience Center, University of Padua, 35128 Padova, Italy

**Keywords:** eating disorders, anorexia nervosa, malnutrition, neuroimaging, fractal dimension, cortical complexity

## Abstract

Fractal Dimension (FD) has shown to be a promising means to describe the morphology of cortical structures across different neurologic and psychiatric conditions, displaying a good sensitivity in capturing atrophy processes. In this study, we aimed at exploring the morphology of cortical areas by means of FD in 58 female patients with Anorexia Nervosa (AN) (38 currently underweight and 20 fully recovered) and 38 healthy controls (HC). All participants underwent high-resolution MRI. Surface extraction was completed using FreeSurfer, FD was computed using the calcFD toolbox. The whole cortex mean FD value was lower in acute AN patients compared to HC (*p* < 0.001). Recovered AN patients did not show differences in the global FD when compared to HC. However, some brain areas showed higher FD in patients than controls, while others showed the opposite pattern. Parietal regions showed lower FD in both AN groups. In acute AN patients, the FD correlated with age (*p* < 0.001), body mass index (*p* = 0.019) and duration of illness (*p* = 0.011). FD seems to represent a feasible method to explore cortical complexity in patients with AN since it demonstrated to be sensitive to the effects of both severity and duration of malnutrition.

## 1. Introduction

Anorexia Nervosa (AN) is a severe psychiatric disorder with a typical onset during adolescence [1], characterized by severe and prolonged alterations of energy intake and high levels of mortality. Although there is a notable interest in understanding the effects of starvation on the brain, a full characterization of brain changes in patients with AN is still at its first stages [2]. The onset of AN typically occurs when neurodevelopment is still ongoing [3] and it is possible that the effects of malnutrition have a different impact in brain areas that are in a sensitive period of growth at the time of AN onset [4]. Moreover, evidence suggests that prenatal and perinatal factors are involved in the pathogenesis of AN and, for this reason, it is not easy to distinguish the structural brain alterations preceding the AN onset from the ones that might be a consequence of the disorder.

Most studies to date employed a Voxel-Based Morphometry approach and found a globally reduced GM volume, but inconsistent results emerged in the identification of specific regional changes in AN [2]. In addition, only few studies found a significant correlation with body weight or amount of weight loss, and almost none with age of onset or duration of illness [5]. The use of a Surface-Based Morphometry approach did not result in more consistent findings. In fact, while some studies reported a correlation between cortical thickness and BMI, others failed in evidencing a relationship between these two parameters [6,7,8,9]. Generally, a reduction of cortical thickness is described in different studies, but the extent of the reduction varies from almost the whole cortex to about a quarter or one third [6,9]. 

Other surface-based methods, such as the local gyrification index and cortical folding, have been employed to describe brain cortical changes in patients with AN [10,11,12]. Both measures displayed significant alterations in acute patients, but the interpretation of these findings is not clear, since gyrification tends to develop early in childhood and its alteration is usually attributed to prenatal or very early insults [13].

A novel way to quantify and analyze the cortex from a morphological and structural point of view has been offered by fractal geometry [14], which is specifically designed for the analysis of complex structural and morphological patterns [15]. The application of fractal geometry to neuroscience is consistent with the evidence, already highlighted by the increasing application of complex network science to neuroimaging data, that the central nervous system is organized in nested and hierarchical organization patterns that need to balance both regularity and randomness [16]. This multi-level structural organization of the brain seems to be well-described by fractal geometry, which is based on the concept of “self-similarity” [17]. Since the fractal properties of the cerebral cortex arise secondarily to folding [18], structural MRI studies used fractal dimension to quantify the morphological complexity of the cortex and its convolutional properties both in clinical and non-clinical samples [19,20,21,22].

Two types of fractal dimension can be considered, depending on whether the volume of the gray matter is included in the computation. Incorporating the volume of the gray matter into the computation ensures that changes in cortical thickness are directly integrated within the fractal dimension estimation. Thus, interestingly, fractal dimension appears to co-vary with both cortical thickness and gyrification [22]. Furthermore, it also appears to demonstrate a great sensitivity to detect cortical atrophy and to describe age-related effects [15,23]. Prior studies have suggested that FD may provide distinct information from the ones that are provided by conventional cortical structural indices such as gyrification, cortical thickness and sulcal morphology, and evidenced the presence of global and regional FD alterations in different psychiatric disorders such as ADHD, schizophrenia, bipolar and obsessive-compulsive disorders [20,21,24,25,26,27]. The use of FD to identify unique characteristics of cortical morphology could be particularly useful in the evaluation of AN, since this disorder has been shown to be characterized by complex (and not entirely determined) patterns of brain morphological alterations. Consistently with this, the first study investigating FD in AN [28] highlighted different patterns of absolute mean curvature and FD alterations in acute AN patients when compared to healthy subjects. More specifically, this research evidenced the presence of a higher FD in the left precentral gyrus as well as a trend toward a lower FD in frontal and occipital areas in patients with AN [28]. 

In the present study, we used a surface-based approach to explore the use of FD to describe cortical complexity in patients with AN. We hypothesize a reduction of cortical complexity in acute underweight patients and an improvement of this feature after remission.

## 2. Methods

Fifty-eight patients with AN (38 with acute AN and 20 in full remission) and 38 HC were included in the study. The sample included was the same as in a previous study [29]. Mean age of the patients was 26.1 years (SD = 7.2) ranging from 15.5 to 40.5 years old. Patients with AN were recruited from the Padova Hospital Eating Disorders Unit. AN was defined according to DSM-5 criteria. All patients fulfilling the inclusion criteria who were in treatment or referred to the Unit while the study was being carried out were asked to participate. A sample of HC similar to the patient group in age, ethnicity, educational level and hand lateralization was recruited from the same geographical area.

Patients who recovered from AN had full AN in their lifetime but have been asymptomatic for at least 6 months prior to the time of scanning [mean remission time: 38.5 months (SD = 33.2); range = 6–96 months]. The main clinical characteristics of the sample are reported in Table 1. Exclusion criteria for the recovered group were bingeing, food restriction, excessive exercise, amenorrhea, fasting and purging in the last 6 months. In the year following the study, none of the recovered patients relapsed. Moreover, recovered patients were within the normal weight range at the time of the scan (BMI ≥ 18.5). Exclusion criteria for all subjects were male gender, history of head trauma or injury with loss of consciousness, history of any serious neurological or medical illness, active use of systemic steroids, pregnancy, active suicidality or major depression, history of substance/alcohol abuse or dependence, bipolar disorder or schizophrenia spectrum disorder, moderate mental impairment (IQ < 60) or learning disabilities, use of medications other than antidepressants and known contraindications to conventional MRI. For healthy women, additional exclusion criteria were a history of any psychiatric disorder and the presence of first-degree relatives with an eating disorder. 

At the time of recruitment, some individuals were excluded from the study: five AN patients were under antipsychotic medication and/or reported severe comorbidity; one AN patient and one healthy subject reported a previous head trauma; one AN patient, three recovered AN and two healthy subjects were not available to undergo MRI scanning when scheduled. The final sample comprised of 96 women (38 with AN, 20 recovered from AN and 38 HC). No further subject was excluded due to problems with scan acquisition, gross brain alterations or motion artifacts.

Ethical permission was obtained from the ethics committee of the Padova Hospital on 10 June 2008 (ID 1598P). After completely describing the study to the subjects, written informed consent was obtained.

A diagnostic interview according to the Eating Disorders Section of the Structured Clinical Interview for DSM-5 [30] was performed in all subjects. A semi-structured interview was also used to collect socio-demographic and clinical variables [29,31]. Depressive and obsessive-compulsive symptoms were assessed by means of the Hopkins Symptoms Checklist [32]. Eating psychopathology was assessed by the Eating Disorders Inventory [33]. The Edinburgh Handedness Inventory [34] was also administered. 

All subjects were recruited at the Eating Disorder Unit of the Hospital of Padova, fulfilled the diagnosis for AN according to DSM-IV criteria and were medically stable at the time of scanning. AN diagnosis was established by experienced senior consultants. Different diagnostic subtypes were observed at the time of scanning: 32 AN patients (84%) were restrictive, six AN patients were of the binge-eating/purging subtype and seven patients who were restrictive at the time of the present study reported previous recurrent binge eating and/or purging. Fourteen AN patients and four recovered women were under treatment with antidepressant drugs at the time the study was conducted (acute AN: one case mirtazapine, two paroxetine, two escitalopram, one fluoxetine, eight sertraline; recovered AN: four sertraline).

### 2.1. MRI Data Acquisition

Data were collected on a Philips Achieva 1.5 Tesla MRI scanner equipped with an eight-channel standard quadrature head coil equipped for echo-planar imaging. A high-resolution three-dimensional (3D) T1-weighted anatomical image was also acquired, in a gradient-echo sequence (repetition-time = 20 s, echo time = 3.78 ms, flip angle = 20°, 160 sagittal slices, acquisition voxel size = 1 × 0.66 × 0.66 mm, field of view 21–22 cm).

### 2.2. Data Processing and Statistics

Data processing was performed using the FreeSurfer package (Martinos Center for Biomedical Imaging, Massachusetts General Hospital, Boston) version 5.3.0 [35]. The preprocessing was carried out according to the standard description using the following steps: skull-stripping and intensity correction, gray matter–white matter boundary determination for each cortical hemisphere using tissue intensity and neighborhood constraints, and finally, tessellation of the resulting surface boundary to generate multiple vertices across the whole brain before inflating.

Surface reconstruction and segmentation were inspected, and minor manual intervention was performed according to FreeSurfer guidelines. After cortical reconstruction, the cortex was parcellated based on individual gyral and sulcal structures [36]. Cortical thickness and local gyrification index were calculated as described in our previous work [10]. 

**Figure 1:**
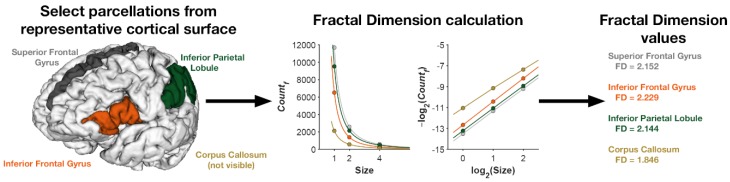
Illustration of the fractal dimension calculation. Individual parcellated regions (or the entire cortical ribbon) are isolated. For each region, the number of voxels at each respective box size is across different spatial resolutions, adjusting for alignment of the ‘boxes’ to the structure using the dilation algorithm. The counts and box sizes are then log-log transformed and the slope calculated. The slope in log-log space is taken as the fractal dimension of the region.

A freely available MATLAB toolbox, calcFD [15] (http://cmadan.github.io/calcFD/), was used to compute the fractal dimension of the cortical ribbon and of parcellated regions of the cortex. The toolbox uses intermediate files generated as part of the standard FreeSurfer analysis pipeline to perform the calculation. Fractal dimension has been shown to be more robust to alignment variability [37] and head motion [38]. We calculated FD of the cortical ribbon (i.e., FD of the filled volume) [15] using the dilation algorithm implemented in the calcFD toolbox and box sizes of 1, 2, and 4. Figure 1 illustrates the fractal dimension calculation for a representative parcellated cortical surface Correlation was performed using Spearmen’s *ρ* (rho), while group comparisons were performed by means of GLM with age and hand-lateralization as covariates of no interest when appropriate. Given that FD values in the different areas of the brain cannot be considered as independent, in order to control for multiple tests, we adjusted for the False Discovery Rate (FDR) [39].

## 3. Results

Table 2 shows the average FD values of the three groups in the whole brain and in the frontal, parietal, temporal and occipital lobes. 

**Figure 2:**
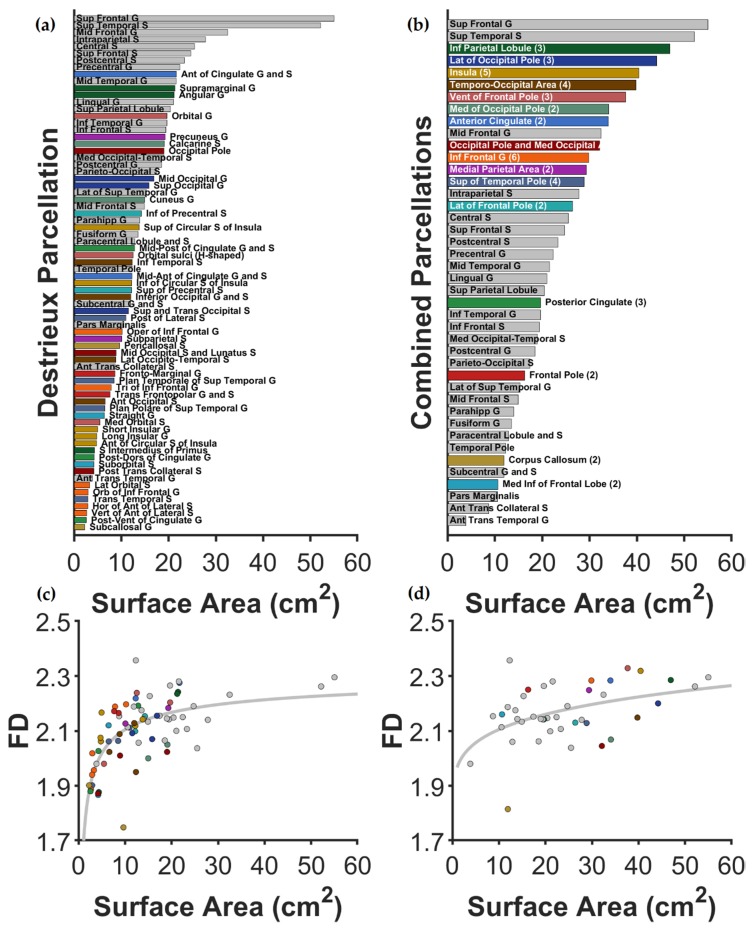
Mean surface area for all parcellation regions using the Destrieux atlas. Figures (**a**,**c**) show the surface area for parcellations in the original Destrieux atlas; Figures (**b**,**d**) show areas for combined regions—where the original regions were considered too small for reliable FD calculations. Regions maintained (not combined) in all plots are shown in gray. Regions that were combined to form larger regions are shown in distinct colors, matched between the panels and in Figure 3C. For Figure (**b**), after each combined region’s name, the number of Destrieux regions combined is included in parentheses. Figures (**c**,**d**) show the relationship between surface area and FD (across all participants), showing that FD becomes increasingly distinct from size for larger regions and that the combined regions increased this property. As above, Figure c corresponds to the original Destrieux atlas, whereas figure d uses the recombined regions.

**Figure 3:**
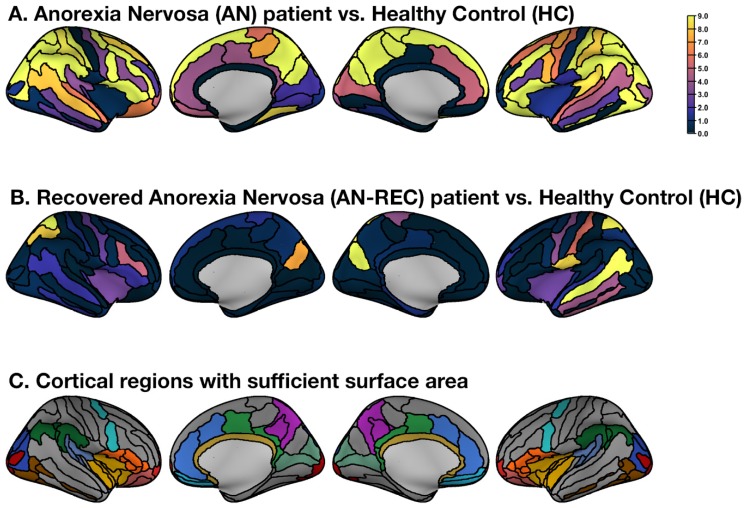
Unthresholded group comparison (F-statistics) plotted on the inflated cortical surface for the combined regions. (**A**) Regional F-statistics comparing Anorexia Nervosa (AN) patients and Healthy Controls (HC). (**B**) Regional F-statistics comparing recovered Anorexia Nervosa (AN-REC) patients with Healthy Controls (HC). (**C**) All cortical regions were included in the analysis. Regions shown in color were recombined with respect to the original Destrieux atlas to yield sufficiently large surfaces areas; colors match the labels and areas shown in Figure 2.

Since the fractal dimension is not sensitive to smaller structures [37,40], we combined small regions with nearby anatomical regions for regions that were insufficient in size. Figure 2 illustrates the area of the original Destrieux parcellations as well as the combined regions. Figure 2 also shows the average surface area for each cortical region and the relationship between area and FD, similar to previous work [37]. As shown in the figure, relatively larger regions have a weak relationship between size and FD, allowing for the shape-related properties of FD to be sensitive to potential group differences (all FD values are included in Tables S1 and S2). Figure 3C shows these regions on the inflated cortical surface for the combined regions.

Patients with acute AN displayed significantly lower FD values in comparison to HC in 22 of the considered cortical areas, as shown in Figure 3A. In both hemispheres, the mean FD value of the cortical ribbon was significantly lower in in inferior frontal, middle frontal, superior frontal, postcentral and superior temporal (lateral aspect) gyri, in paracentral, superior parietal and inferior parietal lobules, in superior frontal, intraparietal, parieto-occipital, postcentral and marginal branch of the cingulate sulci, in the lateral aspect of occipital and frontal poles and in a medial parietal area encompassing the precuneus and the subparietal sulcus. In the left hemisphere only, we additionally found significantly decreased FD values in the precentral gyrus, in the superior aspect of the temporal pole and in a temporal-occipital area encompassing the anterior occipital sulcus, the inferior temporal sulcus, the lateral occipito-temporal sulcus and the inferior occipital gyrus. In the right hemisphere, we found decreased FD values in the medial occipital-temporal sulcus. (FD values are reported in Table S1 in the Supplementary Materials ). No differences in FD values were observed in patients of the restricting type in comparison to those of the binge-eating/purging type (F (3, 34) = 0.005, *p* = 0.946 for total FD), nor in those who were taking antidepressants in comparison to those who did not (F (3, 34) = 0.478, *p* = 0.494, for total FD). 

Recovered AN patients did not show differences in the global and lobar FD when compared to HC. The mean FD value of the cortical ribbon was significantly higher in the AN-REC group when compared to HC in the left superior temporal sulcus and in the left subcentral gyrus and sulcus as shown in Figure 3B (FD values are reported in Table S1). Some areas showed reduced FD in both acute and recovered AN patients in comparison to healthy women: the left and right superior parietal lobule, the left postcentral gyrus, the right intraparietal sulcus and the left and right parieto-occipital sulcus. 

**Figure 4:**
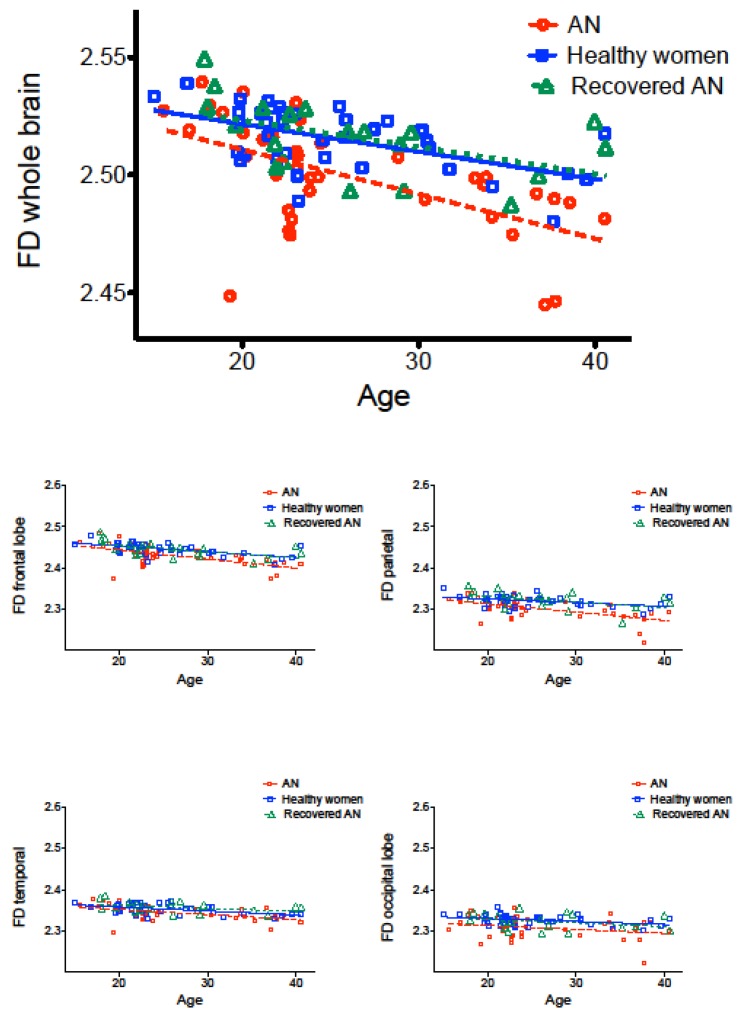
Correlations between whole-brain (cortical ribbon) and lobar FD and age in the three groups.

As expected, FD values were significantly negatively correlated with age in all three groups (Table 3) (compare with [15,38,40]). However, the decline of FD along with age tended to be stronger in the acute AN patients than in the other two groups, especially for the frontal and parietal lobes (Figure 4). In all three groups, no differences were observed in left-handed or mixed-handed (Edinburgh scores between −70 to +70) individuals in comparison to right-handed ones. Table 3 shows the correlations (Spearman’s *ρ* [rho] rank correlation) between FD values and clinical variables in the three groups. Significant positive correlations emerged between whole-brain FD and BMI in acute AN, but not in the recovered group (see also [38] for a normative comparison). The FD value was also significantly negatively correlated with the duration of illness in the acute AN group. A significant negative correlation between the age of onset of the disorder and FD emerged in the recovered group. In this last group, the duration of the recovered status showed a negative nonsignificant correlation with FD (rho = −0.386, *p* = 0.093).

The whole-brain FD positively correlates with the volume of the cortex in all the three groups and with overall local gyrification index in HC, but not in the two AN groups. 

## 4. Discussion

In this paper, we explored the morphological complexity of cortical and subcortical brain structures by means of FD and investigated the relationship between FD with clinical variables. Our results showed the presence of a globally reduced cortical FD in patients with acute AN, while patients who recovered from the disorder did not show alterations in global FD values. This observation, together with the correlation between FD and BMI in the acute AN group, allowed us to hypothesize that a global reduction in cortical complexity may be an effect of malnutrition that can be recovered along with weight recovery. FD has been described in the literature as a sensitive measure to describe cortical atrophy and the effects of aging [15,22,23] and our data show that it also seems to describe the complex patterns of cortical morphology that are secondary to the effects of malnutrition in AN. The cortical structural modifications in AN are likely to depend on many factors, such as malnutrition, dehydration and endocrinological factors, but they could also reflect the alterations of neurodevelopmental trajectories that are hypothesized to precede the onset of the disorder [3]. The hypothesized developmental origin of AN [41] and the effects of its onset in critical developmental phases make it important to consider the relationship between any cortical alterations, the duration of the disorder and the patient’s age and age of onset in the evaluation of structural MRI findings. Our results indicated that both the patient’s age and the duration of the disorder correlate inversely with FD, suggesting an impact of AN on the reduction of cortical complexity. Since cortical complexity, measured by FD, is likely to reduce from adolescence to adulthood as a result of the cortical modeling mechanisms that physiologically occur with aging [42], we hypothesize an impact of the disorder in accelerating this process. 

The observed correlation between FD and gyrification in HC, but not in patients with AN, suggests a sensitivity of FD to the effect of malnutrition. A direct correlation between FD and gyrification has been emphasized by previous studies in healthy as well as in neurological populations, indicating the sensitivity of FD in capturing the role of cortical folding in determining cortical complexity [15,22]. Gyrification appears to be largely determined during the earlier neurodevelopmental phases [13] and alterations in this structural parameter have been already pointed out by previous studies on AN patients [10,12]. The absence of a correlation between these two parameters in AN suggests that, in this group, FD could probably be more susceptible than gyrification to the prolonged effects of malnutrition.

From a regional analysis of cortical areas, we identified an FD reduction in various parietal regions that are crucial for the integration of body-image perception abilities both in acute and in recovered AN patients. These results suggest that these areas may be particularly susceptible to disorder-specific alterations and require to be specifically investigated by further research. Except for these regions, in the recovered group we identified some areas that showed no statistically significant differences in cortical complexity, while others showed a higher FD than HC. These findings could reflect reshaping processes induced by re-nutrition, therefore supporting the role of FD in describing how nutritional processes can influence the brain morphology. It is not clear why in the recovered patients the values of FD displayed different patterns in different brain regions. The negative trend we observed between FD and duration of recovery in this group might indicate that after an initial increase of cortical complexity following weight recovery—which probably implies the occurrence of reparative processes—FD tends to decrease towards average values. It is possible that this process occurs with different trajectories in different areas of the brain. In addition, since we observed a negative correlation between FD and age of onset in the recovered group, it seems that these reparative processes are more evident in patients with an earlier age of onset. 

The present study has several strengths, as well as important limitations. It explores cortical complexity in AN by means of FD, a novel parameter that has been demonstrated to have a good sensitivity to cortical atrophy and age-related brain differences. The evaluation of cortical morphology with FD allows widening the horizons of surface-based cortical analysis, by integrating the information given by cortical thickness and gyrification with novel and non-redundant data. Furthermore, the correlation between FD alterations and the duration of illness is a new and interesting finding about MRI surface-based analysis in AN, hinting at the potentialities of this morphological index in capturing the effects of prolonged starvation on the cortical structure. A limitation of this study is represented by its cross-sectional design. In fact, longitudinal data could be particularly useful to understand how cortical complexity varies with the clinical course of the disorder and with weight recovery. Another limitation can be found in the absence of male patients in the sample. Any inference about alterations in cortical complexity in male patients with AN cannot be made and would be an interesting topic to explore in future studies.

In conclusion, the present study evidences that FD should be considered particularly useful to investigate the morpho-structural properties of brain cortex in AN, since it demonstrated to be suitable for identifying the negative effects of different clinical variables on cortical structure and giving non-redundant information with respect to other surface-based indexes.

## Figures and Tables

**Table 1:** Socio-demographic and clinical characteristics of the sample.

	AN		AN-REC		HC		AN vs. HC	AN-REC. vs. HC
	(*n* = 38)		(*n* = 20)		(*n* = 38)			
	Mean	SD	Mean	SD	Mean	SD	Z (*p*)	Z (*p*)
Age (years)	26.1	7.2	26.3	7.0	25.2	6.7	0.38 (0.701)	0.44 (0.659)
Baseline BMI (kg/m^2^)	16.0	1.8	19.6	1.6	21.6	3.0	7.42 (0.000)	3.09 (0.002)
Lowest BMI (kg/m^2^)	14.0	1.8	15.7	1.4	19.8	2.5	7.17 (0.000)	5.35 (0.000)
Weight loss (kg)	7.1	2.8	5.2	3.1	3.4	1.7	-	-
Age of onset (years)	18.3	5.0	17.7	3.2	-	-	-	-
Duration of illness (months)	78.6	81.2	45.7	65.0	-	-	-	-
Duration of recovery (months)			45.4	47.0	-	-	-	-
Edinburgh laterality index	57.1	37.5	60.0	35.2	55.0	42.0	0.52 (0.603)	0.32 (0.749)
Education (years)	14.2	2.2	14.1	2.6	15.4	2.3	2.63 (0.009)	1.94 (0.053)
Drive to thinness	9.9	6.1	-	-	2.3	4.2	5.492 (0.000)	-
Depression	1.4	0.8	-	-	0.7	0.6	3.844 (0.000)	-
Trait anxiety	56.6	9.7	-	-	39.3	9.6	5.883 (0.000)	-
Cortex volume (mm^3^)	440,936	38,526	456,932	36,916	458,753	31,225	9.55 (0.003)	0.07 (0.80)
Gyrification Index	2.85	0.09	2.90	0.09	2.90	0.11	2.09 (0.04)	0.08 (0.93)
Surface area (mm^2^)	160,640	13,527	157,348	9381	165,082	12,113	2.20 (0.142)	6.11 (0.017)
Cortical Thickness (mm)	2.49	0.12	2.51	0.11	2.48	0.12	0.52 (0.14)	1.05 (0.30)

GLM, including Total Intracranial Volume as a covariate of no interest; BMI = body mass index Clinical assessment and Follow-up.

**Table 2:** Cortical structures FD differences analysis between AN, REC-AN, and HC groups.

	AN	AN-REC	HC	AN vs. HC	AN-REC vs. HC
	Mean (SD)	Mean (SD)	Mean (SD)	F* (*p*)	F* (*p*)
Whole Brain (Cortical Ribbon)	2.49 (0.02)	2.52 (0.02)	2.51 (0.01)	**16.36 (0.000)**	0.32 (0.573)
Frontal Lobe	2.43 (0.02)	2.44 (0.02)	2.44 (0.01)	**13.07 (0.001)**	0.05 (0.829)
Parietal Lobe	2.30 (0.02)	2.32 (0.02)	2.32 (0.01)	**19.75 (0.000)**	0.72 (0.400)
Temporal Lobe	2.34 (0.02)	2.36 (0.01)	2.35 (0.01)	**5.40 (0.023)**	3.46 (0.068)
Occipital Lobe	2.30 (0.02)	2.32 (0.02)	2.32 (0.01)	**15.31 (0.000)**	0.55 (0.462)
Left Superior Parietal Lobule	2.13 (0.06)	2.16 (0.03)	2.19 (0.04)	**26.956 (0.000)**	**8.711 (0.005)**
Right Superior Parietal Lobule	2.11 (0.06)	2.13 (0.04)	2.16 (0.03)	**23.580 (0.000)**	**9.600 (0.003)**
Left Postcentral Gyrus	2.06 (0.06)	2.07 (0.04)	2.10 (0.04)	**9.851 (0.002)**	**5.689 (0.021)**
Right Intraparietal Sulcus	2.11 (0.05)	2.12 (0.04)	2.15 (0.03)	**18.715 (0.000)**	**8.170 (0.006)**
Left Parieto-Occipital Sulcus	2.13 (0.04)	2.13 (0.04)	2.16 (0.02)	**12.640 (0.001)**	**10.339 (0.002)**
Right Parieto-Occipital Sulcus	2.15 (0.04)	2.16 (0.04)	2.18 (0.03)	**17.123 (0.000)**	**7.250 (0.009)**

* F (GLM with age and hand lateralization as covariates of no interest; degrees of freedom = 3), *p* threshold determined based on FDR < 0.025. Significant effects are highlighted in **bold**.

**Table 3:** Correlation between whole-brain (cortical ribbon) FD and clinical variables within each group.

	AN (*n* = 38) Rho (*p*)	AN-REC (*n* = 20) Rho (*p*)	Healthy Controls (*n* = 38) Rho (*p*)
	**Whole-brain FD**		
Age	−0.608 (0.000) *	−0.617 (0.004) *	−0.527 (0.001) *
Body mass index (BMI)	0.380 (0.019) *	−0.351 (0.130)	−0.209 (0.207)
Duration of illness	−0.406 (0.011) *	−0.111 (0.642)	
Age of AN onset	−0.265 (0.108)	−0.586 (0.007) *	
Cortical volume	0.638 (0.000) *	0.537 (0.015) *	0.496 (0.002) *
Cortical gyrification	0.258 (0.118)	0.376 (0.102)	0.514 (0.001) *
Cortical thickness	0.000 (0.998)	0.65 (0.787)	0.025 (0.883)

FD: Fractal Dimension; Spearman’s *ρ(rho)*; * FDR < 0.025.

## Supplementary Materials

The following are available online at https://www.mdpi.com/2077-0383/9/3/833/s1, Table S1: FD differences analysis for combined cortical regions, comparing the AN, REC-AN and HC groups, Table S2: FD differences analysis for all cortical regions of the Destrieux atlas, comparing the AN, REC-AN and HC groups.
