# Supplementary material for: Cortical Complexity in Anorexia Nervosa: A Fractal Dimension Analysis
Source: J Clin Med. 2020 Mar 19;9(3):833. doi: 10.3390/jcm9030833 (PMC7141241; doi:10.3390/jcm9030833)
Supplement: Supplementary file 1 [file jcm-09-00833-s001.pdf]

Table S1. FD differences analysis for combined cortical regions, comparing the AN, REC-AN, and HC groups.

	Lat	AN<br>Mean (SD)	AN-REC<br>Mean (SD)	HC<br>Mean (SD)	AN vs HC<br>F* (p)	AN-REC vs HC<br>F* (p)
Frontal pole	L	2.25 (0.04)	2.26 (0.03)	2.25 (0.03)	0.655 (0.421)	2.503 (0.119)
	R	2.24 (0.04)	2.25 (0.04)	2.25 (0.02)	4.978 (0.029)	0.558 (0.458)
Paracentral lobule and S	L	2.05 (0.06)	2.07 (0.04)	2.09 (0.04)	**11.040 (0.001)**	3.733 (0.059)
	R	2.04 (0.04)	2.05 (0.06)	2.06 (0.04)	**5.790 (0.019)**	1.409 (0.240)
Subcentral G and S	L	2.19 (0.03)	2.22 (0.03)	2.20 (0.02)	1.611 (0.208)	8.289 (0.006)
	R	2.17 (0.04)	2.17 (0.04)	2.17 (0.03)	0.571 (0.452)	0.462 (0.500)
Anterior cingulate G and S	L	2.27 (0.04)	2.28 (0.03)	2.28 (0.02)	4.947 (0.029)	0.111 (0.741)
	R	2.28 (0.03)	2.29 (0.02)	2.30 (0.02)	3.947 (0.051)	0.018 (0.894)
Posterior cingulate G and S	L	2.13 (0.04)	2.13 (0.02)	2.14 (0.03)	0.172 (0.679)	0.781 (0.381)
	R	2.14 (0.03)	2.15 (0.04)	2.16 (0.03)	4.133 (0.045)	0.147 (0.703)
Medial aspect of occipital pole	L	2.06 (0.05)	2.08 (0.04)	2.08 (0.03)	5.069 (0.027)	0.292 (0.591)
	R	2.05 (0.05)	2.07 (0.04)	2.07 (0.04)	1.864 (0.176)	0.001 (0.970)
Inferior frontal gyrus	L	2.27 (0.04)	2.29 (0.02)	2.29 (0.02)	**14.220 (0.000)**	0.274 (0.602)
	R	2.27 (0.03)	2.29 (0.03)	2.29 (0.02)	**10.495 (0.002)**	1.713 (0.196)
Middle frontal G	L	2.22 (0.04)	2.24 (0.04)	2.25 (0.03)	**10.667 (0.002)**	0.005 (0.943)
	R	2.21 (0.04)	2.23 (0.03)	2.24 (0.02)	**14.047 (0.000)**	0.182 (0.671)
Superior frontal G	L	2.29 (0.04)	2.31 (0.03)	2.31 (0.02)	**10.440 (0.002)**	0.001 (0.978)
	R	2.27 (0.04)	2.29 (0.03)	2.30 (0.02)	**19.931 (0.000)**	1.069 (0.306)
Fusiform G	L	2.17 (0.04)	2.17 (0.3)	2.18 (0.02)	0.356 (0.552)	0.909 (0.345)

	R	2.18 (0.04)	2.18 (0.03)	2.17 (0.04)	1.384 (0.243)	0.627 (0.432)
Lingual G	L	2.10 (0.04)	2.10 (0.04)	2.10 (0.04)	0.151 (0.699)	0.243 (0.624)
	R	2.09 (0.04)	2.10 (0.04)	2.10 (0.03)	1.934 (0.169)	0.136 (0.713)
Parahippocampal G	L	2.14 (0.04)	2.15 (0.04)	2.14 (0.03)	0.051 (0.821)	1.222 (0.274)
	R	2.14 (0.05)	2.15 (0.05)	2.14 (0.04)	0.009 (0.925)	0.294 (0.590)
Superior parietal lobule	L	2.13 (0.06)	2.16 (0.03)	2.19 (0.04)	**26.965 (0.000)**	**8.711 (0.005)**
	R	2.11 (0.06)	2.13 (0.04)	2.16 (0.03)	**23.580 (0.000)**	**9.600 (0.003)**
Postcentral G	L	2.06 (0.06)	2.07 (0.05)	2.10 (0.04)	**9.851 (0.002)**	5.689 (0.021)
	R	2.02 (0.06)	2.05 (0.05)	2.07 (0.04)	**13.248 (0.001)**	1.195 (0.279)
Precentral G	L	2.14 (0.06)	2.15 (0.03)	2.17 (0.04)	6.035 (0.016)	3.568 (0.064)
	R	2.14 (0.06)	2.14 (0.04)	2.16 (0.03)	3.252 (0.076)	2.570 (0.115)
Medial parietal area	L	2.23 (0.03)	2.26 (0.03)	2.25 (0.02)	**13.483 (0.000)**	0.576 (0.451)
	R	2.23 (0.03)	2.26 (0.04)	2.26 (0.02)	**19.828 (0.000)**	0.808 (0.373)
Anterior transverse temporal G	L	1.99 (0.05)	1.98 (0.04)	2.00 (0.04)	0.057 (0.812)	1.079 (0.303)
	R	1.97 (0.05)	1.96 (0.03)	1.98 (0.04)	2.495 (0.119)	1.846 (0.180)
Lateral aspect of superior temporal G	L	2.21 (0.04)	2.23 (0.02)	2.23 (0.02)	**5.517 (0.022)**	1.399 (0.242)
	R	2.22 (0.04)	2.23 (0.02)	2.24 (0.03)	**5.951 (0.017)**	0.028 (0.868)
Inferior temporal G	L	2.26 (0.03)	2.28 (0.03)	2.26 (0.03)	0.017 (0.896)	4.483 (0.039)
	R	2.25 (0.02)	2.27 (0.03)	2.26 (0.02)	2.412 (0.125)	1.481 (0.229)
Middle temporal G	L	2.26 (0.03)	2.29 (0.03)	2.27 (0.03)	2.293 (0.134)	4.010 (0.050)
	R	2.28 (0.02)	2.29 (0.02)	2.29 (0.02)	2.871 (0.094)	0.257 (0.614)
Occipital pole and medial occipital area	L	2.01 (0.05)	2.03 (0.03)	2.03 (0.03)	3.894 (0.052)	0.032 (0.858)

	R	2.06 (0.05)	2.07 (0.03)	2.07 (0.04)	1.188 (0.279)	0.480 (0.491)
Temporal pole	L	2.36 (0.04)	2.36 (0.03)	2.36 (0.03)	0.225 (0.637)	0.078 (0.781)
	R	2.35 (0.04)	2.35 (0.04)	2.36 (0.04)	0.127 (0.723)	0.661 (0.420)
Central sulcus	L	2.04 (0.04)	2.04 (0.04)	2.05 (0.03)	0.703 (0.404)	0.482 (0.491)
	R	2.03 (0.06)	2.03 (0.04)	2.04 (0.05)	0.057 (0.812)	0.070 (0.792)
Pars marginalis	L	2.08 (0.03)	2.11 (0.03)	2.11 (0.03)	**17.337 (0.000)**	0.080 (0.778)
	R	2.11 (0.04)	2.13 (0.04)	2.14 (0.04)	**7.313 (0.009)**	0.609 (0.439)
Insula	L	2.31 (0.03)	2.33 (0.02)	2.32 (0.02)	1.287 (0.260)	2.692 (0.107)
	R	2.31 (0.03)	2.32 (0.02)	2.32 (0.02)	0.495 (0.484)	2.909 (0.094)
Anterior transverse collateral S	L	2.15 (0.05)	2.16 (0.04)	2.17 (0.05)	2.797 (0.099)	0.245 (0.623)
	R	2.14 (0.05)	2.15 (0.04)	2.15 (0.03)	1.465 (0.230)	0.024 (0.877)
Inferior frontal S	L	2.14 (0.03)	2.16 (0.03)	2.16 (0.05)	2.736 (0.102)	0.040 (0.842)
	R	2.11 (0.05)	2.15 (0.03)	2.13 (0.04)	3.509 (0.065)	4.872 (0.032)
Middle frontal S	L	2.11 (0.06)	2.13 (0.05)	2.12 (0.05)	0.571 (0.452)	1.479 (0.229)
	R	2.14 (0.05)	2.15 (0.05)	2.15 (0.04)	2.279 (0.135)	0.264 (0.609)
Superior frontal S	L	2.18 (0.06)	2.19 (0.03)	2.20 (0.03)	**5.970 (0.017)**	0.643 (0.426)
	R	2.17 (0.05)	2.20 (0.03)	2.20 (0.03)	**8.190 (0.006)**	0.654 (0.422)
Inferior parietal lobule	L	2.26 (0.03)	2.29 (0.03)	2.29 (0.02)	**26.290 (0.000)**	0.372 (0.544)
	R	2.27 (0.04)	2.30 (0.03)	2.30 (0.02)	**24.368 (0.000)**	0.327 (0.570)
Intraparietal S	L	2.13 (0.03)	2.16 (0.02)	2.17 (0.04)	**13.067 (0.000)**	0.976 (0.328)
	R	2.11 (0.05)	2.12 (0.04)	2.15 (0.03)	**18.715 (0.000)**	8.170 (0.006)
Lateral aspect of occipital pole	L	2.17 (0.04)	2.20 (0.03)	2.20 (0.03)	**13.531 (0.000)**	0.274 (0.603)

	R	2.19 (0.04)	2.23 (0.04)	2.22 (0.02)	**21.324 (0.000)**	0.265 (0.609)
Temporal-occipital area	L	2.12 (0.03)	2.14 (0.03)	2.14 (0.03)	**9.364 (0.003)**	0.226 (0.637)
	R	2.13 (0.03)	2.14 (0.03)	2.22 (0.02)	0.644 (0.425)	0.565 (0.455)
Medial occipital-temporal S	L	2.14 (0.04)	2.15 (0.02)	2.15 (0.03)	1.278 (0.262)	0.056 (0.813)
	R	2.13 (0.04)	2.14 (0.03)	2.15 (0.02)	8.453 (0.005)	0.359 (0.551)
Ventral aspect of frontal pole	L	2.31 (0.03)	2.33 (0.02)	2.33 (0.02)	**9.172 (0.003)**	0.029 (0.866)
	R	2.32 (0.03)	2.34 (0.03)	2.34 (0.02)	**6.420 (0.013)**	0.000 (0.999)
Parieto-occipital S	L	2.13 (0.04)	2.13 (0.04)	2.16 (0.02)	**12.640 (0.001)**	**10.339 (0.002)**
	R	2.15 (0.04)	2.16 (0.04)	2.18 (0.03)	**17.123 (0.000)**	**7.250 (0.009)**
Corpus callosum	L	1.76 (0.08)	1.76 (0.06)	1.77 (0.07)	0.309 (0.580)	0.175 (0.678)
	R	1.87 (0.05)	1.86 (0.05)	1.87 (0.05)	0.001 (0.976)	0.018 (0.732)
Postcentral S	L	2.11 (0.04)	2.13 (0.05)	2.14 (0.03)	**8.076 (0.006)**	0.021 (0.886)
	R	2.07 (0.04)	2.09 (0.05)	2.10 (0.05)	**8.613 (0.004)**	0.073 (0.788)
Lateral aspect of frontal pole	L	2.12 (0.04)	2.13 (0.03)	2.14 (0.03)	**7.412 (0.008)**	0.590 (0.446)
	R	2.12 (0.04)	2.14 (0.03)	2.15 (0.03)	**11.441 (0.001)**	0.450 (0.505)
Medial inferior aspect of frontal lobe	L	2.19 (0.03)	2.19 (0.03)	2.19 (0.03)	0.120 (0.731)	0.122 (0.728)
	R	2.11 (0.04)	2.13 (0.04)	2.15 (0.03)	4.546 (0.036)	0.036 (0.850)
Superior temporal S	L	2.24 (0.03)	2.27 (0.02)	2.26 (0.02)	4.441 (0.039)	9.040 (0.004)
	R	2.25 (0.03)	2.28 (0.03)	2.27 (0.02)	**7.832 (0.007)**	1.627 (0.208)
Superior aspect of temporal pole	L	2.12 (0.03)	2.13 (0.02)	2.14 (0.03)	**8.325 (0.005)**	0.150 (0.700)
	R	2.12 (0.03)	2.13 (0.02)	2.13 (0.02)	3.427 (0.068)	0.007 (0.934)

* F (GLM with age and hand lateralization as covariates of no interest; degrees of freedom=3), p threshold determined based on FDR < 0.025. Significant effects that are consistent across both hemispheres are highlighted in **bold**.

Table S2. FD differences analysis for all cortical regions of the Destrieux atlas, comparing the AN, REC-AN, and HC groups

	Lat	AN<br>Mean (SD)	AN-REC<br>Mean (SD)	HC<br>Mean (SD)	AN vs HC<br>F* (p)	AN-REC vs HC<br>F* (p)
Fronto-marginal G	L	2.17 (0.04)	2.19 (0.03)	2.18 (0.03)	1.490 (0.224)	2.301 (0.088)
	R	2.15 (0.04)	2.15 (0.05)	2.15 (0.03)	4.369 (0.007)	2.610 (0.061)
Inferior occipital G and S	L	2.11 (0.06)	2.12 (0.05)	2.13 (0.04)	2.795 (0.046)	2.207 (0.098)
	R	2.13 (0.04)	2.14 (0.04)	2.14 (0.04)	0.492 (0.689)	0.131 (0.942)
Paracentral lobule and S	L	2.05 (0.06)	2.07 (0.04)	2.09 (0.04)	**6.687 (0.000)**	2.462 (0.072)
	R	2.03 (0.06)	2.04 (0.06)	2.06 (0.04)	**4.104 (0.010)**	0.955 (0.420)
Subcentral G and S	L	2.19 (0.03)	2.22 (0.03)	2.20 (0.03)	2.493 (0.067)	3.254 (0.029)
	R	2.17 (0.03)	2.17 (0.04)	2.18 (0.03)	1.941 (0.131)	0.736 (0.535)
Transverse frontopolar G and S	L	2.14 (0.04)	2.17 (0.04)	2.16 (0.03)	2.542 (0.063)	0.715 (0.547)
	R	2.18 (0.04)	2.18 (0.04)	2.20 (0.03)	4.144 (0.009)	1.161 (0.333)
Anterior part of the cingulate G and S (ACC)	L	2.26 (0.04)	2.28 (0.02)	2.28 (0.02)	**4.271 (0.008)**	4.319 (0.008)
	R	2.27 (0.03)	2.28 (0.03)	2.28 (0.02)	**5.114 (0.003)**	1.985 (0.127)
Middle-anterior part of the cingulate G and S	L	2.20 (0.04)	2.21 (0.04)	2.21 (0.03)	2.741 (0.049)	3.690 (0.017)
	R	2.22 (0.03)	2.23 (0.03)	2.24 (0.02)	2.698 (0.052)	1.403 (0.252)
Middle-posterior part of the cingulate G and S	L	2.18 (0.03)	2.19 (0.02)	2.20 (0.02)	1.932 (0.132)	0.487 (0.693)
	R	2.18 (0.03)	2.20 (0.03)	2.20 (0.03)	2.324 (0.082)	2.871 (0.045)
Posterior-dorsal part of the cingulate G	L	2.06 (0.05)	2.04 (0.04)	2.05 (0.04)	0.855 (0.469)	1.284 (0.289)
	R	1.93 (0.06)	2.03 (0.06)	2.05 (0.05)	3.456 (0.021)	1.098 (0.358)
Posterior-ventral part of the cingulate G	L	1.80 (0.07)	1.80 (0.07)	1.81 (0.07)	0.862 (0.465)	2.108 (0.110)

	R	1.95 (0.06)	1.95 (0.08)	1.97 (0.06)	2.805 (0.046)	3.470 (0.022)
Cuneus	L	1.96 (0.06)	1.97 (0.05)	1.98 (0.05)	2.443 (0.071)	0.983 (0.408)
	R	2.18 (0.03)	1.96 (0.04)	1.95 (0.05)	1.222 (0.308)	1.059 (0.374)
Opercular part of the inferior frontal G	L	2.19 (0.03)	2.20 (0.03)	2.21 (0.03)	**7.015 (0.000)**	2.057 (0.117)
	R	2.18 (0.03)	2.20 (0.04)	2.20 (0.03)	**9.815 (0.000)**	4.362 (0.008)
Orbital part of the inferior frontal G	L	2.00 (0.05)	1.99 (0.06)	2.00 (0.05)	3.133 (0.031)	1.354 (0.267)
	R	2.02 (0.06)	2.05 (0.04)	2.05 (0.05)	2.159 (0.100)	0.165 (0.919)
Triangular part of the inferior frontal G	L	2.18 (0.03)	2.20 (0.03)	2.20 (0.02)	**4.424 (0.007)**	0.101 (0.959)
	R	2.17 (0.03)	2.18 (0.03)	2.20 (0.03)	**4.312 (0.007)**	1.948 (0.133)
Middle frontal G	L	2.22 (0.04)	2.24 (0.04)	2.25 (0.03)	**6.979 (0.000)**	2.486 (0.070)
	R	2.21 (0.04)	2.23 (0.03)	2.24 (0.02)	**12.078 (0.000)**	4.941 (0.004)
Superior frontal G	L	2.29 (0.04)	2.31 (0.03)	2.31 (0.02)	**15.052 (0.000)**	**6.978 (0.000)**
	R	2.27 (0.04)	2.29 (0.03)	2.30 (0.02)	**18.568 (0.000)**	**8.435 (0.000)**
Long insular G and central S of the insula	L	2.04 (0.05)	2.05 (0.04)	2.03 (0.04)	1.940 (0.131)	2.015 (0.123)
	R	2.09 (0.04)	2.08 (0.04)	2.08 (0.05)	3.544 (0.019)	1.628 (0.194)
Short insular G	L	2.18 (0.03)	2.19 (0.04)	2.18 (0.03)	0.677 (0.569)	1.310 (0.281)
	R	2.15 (0.03)	2.15 (0.03)	2.15 (0.03)	1.002 (0.397)	0.634 (0.596)
Middle occipital G	L	2.13 (0.04)	2.14 (0.03)	2.16 (0.03)	**9.541 (0.000)**	4.336 (0.008)
	R	2.15 (0.04)	2.18 (0.04)	2.17 (0.03)	**4.266 (0.008)**	1.886 (0.143)
Superior occipital G	L	2.05 (0.05)	2.07 (0.04)	2.08 (0.03)	**3.507 (0.020)**	0.135 (0.939)
	R	2.05 (0.05)	2.08 (0.04)	2.09 (0.04)	**6.270 (0.001)**	0.263 (0.852)
Fusiform G	L	2.17 (0.05)	2.17 (0.02)	2.18 (0.03)	2.118 (0.105)	1.010 (0.395)

	R	2.18 (0.04)	2.18 (0.03)	2.17 (0.04)	1.986 (0.124)	0.415 (0.743)
Lingual G	L	2.10 (0.06)	2.11 (0.04)	2.10 (0.04)	1.987 (0.124)	1.626 (0.194)
	R	2.09 (0.04)	2.10 (0.04)	2.10 (0.03)	1.790 (0.157)	0.349 (0.790)
Parahippocampal G	L	2.14 (0.04)	2.15 (0.04)	2.14 (0.03)	2.011 (0.120)	0.546 (0.653)
	R	2.14 (0.05)	2.15 (0.06)	2.14 (0.04)	1.399 (0.250)	0.220 (0.882)
Orbital G	L	2.18 (0.03)	2.20 (0.03)	2.21 (0.02)	**13.479 (0.000)**	**4.458 (0.007)**
	R	2.20 (0.03)	2.21 (0.04)	2.22 (0.02)	**12.647 (0.000)**	**7.127 (0.000)**
Angular G	L	2.21 (0.04)	2.23 (0.04)	2.24 (0.02)	**8.833 (0.000)**	2.884 (0.044)
	R	2.22 (0.04)	2.25 (0.03)	2.26 (0.03)	**11.482 (0.000)**	3.044 (0.037)
Supramarginal G	L	2.23 (0.04)	2.24 (0.03)	2.25 (0.03)	**10.545 (0.000)**	1.761 (0.166)
	R	2.23 (0.04)	2.25 (0.03)	2.25 (0.02)	**11.160 (0.000)**	**6.868 (0.001)**
Superior parietal G	L	2.13 (0.06)	2.16 (0.03)	2.19 (0.04)	**14.446 (0.000)**	**6.237 (0.001)**
	R	2.11 (0.06)	2.14 (0.04)	2.16 (0.03)	**11.691 (0.000)**	5.401 (0.003)
Postcentral G	L	2.06 (0.06)	2.07 (0.05)	2.10 (0.04)	**4.804 (0.004)**	2.506 (0.069)
	R	2.03 (0.06)	2.06 (0.05)	2.07 (0.04)	**6.744 (0.000)**	1.712 (0.175)
Precentral G	L	2.14 (0.06)	2.15 (0.03)	2.17 (0.04)	**6.910 (0.000)**	**4.384 (0.008)**
	R	2.14 (0.06)	2.14 (0.04)	2.16 (0.03)	**7.041 (0.000)**	**3.754 (0.016)**
Precuneus G	L	2.17 (0.03)	2.19 (0.03)	2.20 (0.02)	**8.176 (0.000)**	3.280 (0.028)
	R	2.16 (0.04)	2.19 (0.03)	2.19 (0.03)	**7.270 (0.000)**	1.145 (0.339)
Straight G	L	2.14 (0.04)	2.14 (0.03)	2.15 (0.03)	**3.508 (0.020)**	0.989 (0.405)
	R	2.08 (0.04)	2.11 (0.05)	2.10 (0.04)	1.949 (0.129)	1.124 (0.348)
Subcallosal G	L	1.87 (0.11)	1.86 (0.12)	1.90 (0.12)	1.646 (0.186)	0.588 (0.625)

	R	1.92 (0.09)	1.92 (0.08)	1.94 (0.08)	0.320 (0.811)	0.778 (0.512)
Anterior tranverse temporal gyrus	L	1.99 (0.05)	1.98 (0.04)	2.00 (0.04)	0.989 (0.403)	2.657 (0.058)
	R	1.96 (0.05)	1.97 (0.03)	1.98 (0.04)	2.359 (0.079)	1.942 (0.134)
Lateral superior temporal G	L	2.21 (0.04)	2.23 (0.03)	2.23 (0.02)	**6.992 (0.000)**	2.019 (0.122)
	R	2.22 (0.04)	2.23 (0.02)	2.24 (0.03)	**6.936 (0.000)**	1.201 (0.318)
Planum polare of the superior temporal G	L	2.04 (0.05)	2.07 (0.05)	2.06 (0.06)	0.900 (0.446)	0.452 (0.717)
	R	2.06 (0.05)	2.07 (0.04)	2.07 (0.05)	0.315 (0.815)	0.353 (0.787)
Temporal plane of the superior temporal G	L	2.07 (0.05)	2.08 (0.04)	2.08 (0.05)	1.345 (0.267)	0.499 (0.685)
	R	2.04 (0.04)	2.06 (0.03)	2.05 (0.05)	0.972 (0.411)	0.548 (0.652)
Inferior temporal G	L	2.26 (0.03)	2.28 (0.03)	2.26 (0.03)	2.578 (0.060)	2.477 (0.071)
	R	2.26 (0.02)	2.27 (0.03)	2.26 (0.02)	3.940 (0.012)	1.813 (0.156)
Middle temporal G	L	2.26 (0.04)	2.28 (0.02)	2.27 (0.03)	**4.774 (0.004)**	3.180 (0.031)
	R	2.28 (0.02)	2.30 (0.02)	2.29 (0.02)	**5.290 (0.002)**	4.125 (0.010)
Horizontal ramus (or segment) of the lateral S	L	1.91 (0.07)	1.94 (0.06)	1.93 (0.04)	0.532 (0.662)	0.217 (0.885)
	R	1.94 (0.04)	1.96 (0.04)	1.96 (0.04)	2.569 (0.061)	0.149 (0.930)
Vertical ramus (or segment) of the lateral S	L	1.88 (0.08)	1.90 (0.07)	1.90 (0.07)	4.960 (0.003)	1.084 (0.364)
	R	1.87 (0.10)	1.89 (0.06)	1.88 (0.06)	0.671 (0.573)	0.266 (0.850)
Posterior ramus (or segment) of the lateral S	L	2.08 (0.03)	2.10 (0.02)	2.11 (0.03)	**5.590 (0.002)**	1.748 (0.168)
	R	2.12 (0.04)	2.13 (0.03)	2.13 (0.03)	**3.478 (0.020)**	0.651 (0.586)
Occipital pole	L	2.00 (0.05)	2.01 (0.05)	2.01 (0.03)	0.614 (0.608)	0.429 (0.733)
	R	2.04 (0.05)	2.04 (0.04)	2.04 (0.04)	0.368 (0.777)	0.941 (0.427)
Temporal pole	L	2.36 (0.04)	2.36 (0.03)	2.36 (0.03)	0.577 (0.632)	2.306 (0.087)

	R	2.35 (0.04)	2.35 (0.04)	2.36 (0.04)	0.123 (0.946)	0.258 (0.855)
Calcarine S	L	2.04 (0.04)	2.05 (0.04)	2.07 (0.03)	**3.953 (0.011)**	2.616 (0.060)
	R	2.04 (0.04)	2.04 (0.04)	2.06 (0.04)	**3.881 (0.012)**	1.472 (0.232)
Central S	L	2.04 (0.05)	2.04 (0.04)	2.05 (0.03)	1.222 (0.308)	0.352 (0.788)
	R	2.03 (0.06)	2.03 (0.06)	2.03 (0.05)	1.974 (0.126)	0.653 (0.585)
Marginal part of the cingulate S	L	2.08 (0.03)	2.11 (0.03)	2.11 (0.03)	**6.609 (0.001)**	1.476 (0.231)
	R	2.11 (0.04)	2.13 (0.04)	2.14 (0.04)	**3.387 (0.023)**	0.406 (0.749)
Anterior segment of the circular S of the insula	L	2.06 (0.04)	2.06 (0.05)	2.07 (0.04)	2.730 (0.050)	1.574 (0.206)
	R	2.08 (0.04)	2.09 (0.06)	2.09 (0.04)	0.121 (0.947)	2.920 (0.042)
Inferior segment of the circular S of the insula	L	2.11 (0.04)	2.12 (0.03)	2.12 (0.03)	0.584 (0.628)	0.055 (0.983)
	R	2.12 (0.04)	2.12 (0.05)	2.12 (0.03)	0.641 (0.591)	0.365 (0.778)
Superior segment of the circular S of the insula	L	2.14 (0.03)	2.16 (0.03)	2.15 (0.02)	0.963 (0.415)	0.904 (0.445)
	R	2.12 (0.04)	2.14 (0.03)	2.14 (0.03)	2.058 (0.113)	0.550 (0.650)
Anterior transverse collateral S	L	2.15 (0.05)	2.16 (0.04)	2.17 (0.05)	1.971 (0.126)	0.324 (0.808)
	R	2.14 (0.05)	2.15 (0.04)	2.15 (0.03)	1.272 (0.290)	0.424 (0.736)
Posterior tranverse collateral S	L	1.83 (0.09)	1.88 (0.07)	1.88 (0.07)	2.901 (0.041)	0.164 (0.920)
	R	1.84 (0.09)	1.89 (0.12)	1.91 (0.11)	3.895 (0.012)	0.671 (0.574)
Inferior frontal S	L	2.14 (0.04)	2.16 (0.03)	2.16 (0.05)	2.583 (0.060)	1.935 (0.135)
	R	2.11 (0.05)	2.15 (0.04)	2.13 (0.04)	1.587 (0.200)	4.553 (0.006)
Middle frontal S	L	2.11 (0.06)	2.13 (0.05)	2.12 (0.05)	1.445 (0.237)	0.705 (0.553)
	R	2.14 (0.05)	2.15 (0.05)	2.15 (0.04)	1.083 (0.362)	0.217 (0.884)
Superior frontal S	L	2.18 (0.04)	2.19 (0.04)	2.20 (0.03)	**7.316 (0.000)**	3.755 (0.016)

	R	2.17 (0.04)	2.20 (0.03)	2.20 (0.03)	**5.584 (0.002)**	1.980 (0.128)
Sulcus intermedius primus (of Jensen)	L	1.89 (0.09)	1.94 (0.07)	1.89 (0.13)	0.450 (0.718)	0.984 (0.407)
	R	1.84 (0.09)	1.85 (0.14)	1.85 (0.09)	1.025 (0.387)	1.562 (0.209)
Intraparietal S and transverse parietal sulci	L	2.13 (0.05)	2.16 (0.03)	2.17 (0.04)	**5.703 (0.001)**	0.974 (0.412)
	R	2.11 (0.05)	2.12 (0.04)	2.15 (0.03)	**10.137 (0.000)**	7.737 (0.000)
Middle occipital S and lunatus S	L	1.99 (0.07)	2.01 (0.05)	2.02 (0.05)	1.870 (0.142)	1.221 (0.311)
	R	2.00 (0.07)	2.03 (0.04)	2.01 (0.07)	0.941 (0.426)	1.148 (0.338)
Sup. and transverse occipital S	L	2.07 (0.04)	2.11 (0.04)	2.09 (0.05)	1.146 (0.337)	2.028 (0.121)
	R	2.07 (0.05)	2.10 (0.05)	2.11 (0.04)	**3.517 (0.019)**	0.074 (0.974)
Anterior occipital S	L	2.00 (0.09)	2.03 (0.08)	2.04 (0.06)	**3.915 (0.012)**	0.505 (0.681)
	R	2.01 (0.08)	2.02 (0.06)	2.04 (0.07)	1.615 (0.193)	1.131 (0.345)
Lateral occipito-temporal S	L	2.07 (0.04)	2.09 (0.05)	2.09 (0.05)	0.341 (0.795)	0.611 (0.611)
	R	2.09 (0.05)	2.10 (0.05)	2.09 (0.05)	0.311 (0.817)	2.139 (0.106)
Collateral and Lingual S	L	2.14 (0.04)	2.15 (0.03)	2.15 (0.03)	0.857 (0.467)	0.584 (0.628)
	R	2.13 (0.04)	2.15 (0.03)	2.15 (0.03)	3.567 (0.018)	1.478 (0.231)
Lateral orbital S	L	1.94 (0.07)	1.95 (0.04)	1.95 (0.06)	0.519 (0.670)	0.170 (0.916)
	R	1.95 (0.06)	1.97 (0.06)	1.98 (0.06)	1.875 (0.142)	1.529 (0.217)
Medial orbital S	L	1.97 (0.05)	1.98 (0.05)	1.98 (0.05)	0.930 (0.431)	0.876 (0.459)
	R	1.98 (0.04)	1.98 (0.04)	1.99 (0.03)	0.586 (0.626)	1.416 (0.248)
Orbital sulci (H-shaped sulci)	L	2.23 (0.04)	2.25 (0.03)	2.24 (0.03)	0.995 (0.400)	0.881 (0.457)
	R	2.22 (0.04)	2.25 (0.05)	2.24 (0.03)	3.151 (0.030)	1.367 (0.263)
Parieto-occipital S	L	2.13 (0.04)	2.14 (0.04)	2.16 (0.02)	**4.684 (0.005)**	3.567 (0.020)

	R	2.15 (0.04)	2.16 (0.04)	2.19 (0.03)	**6.125 (0.001)**	3.196 (0.031)
Pericallosal S	L	1.68 (0.07)	1.68 (0.05)	1.67 (0.08)	0.789 (0.504)	0.343 (0.794)
	R	1.82 (0.04)	1.82 (0.06)	1.82 (0.06)	1.047 (0.377)	1.654 (0.188)
Postcentral S	L	2.11 (0.04)	2.13 (0.05)	2.14 (0.03)	8.192 (0.000)	3.074 (0.035)
	R	2.07 (0.04)	2.09 (0.05)	2.10 (0.05)	3.086 (0.033)	0.310 (0.818)
Inferior part of the precentral S	L	2.13 (0.04)	2.15 (0.03)	2.15 (0.03)	1.781 (0.158)	0.056 (0.982)
	R	2.15 (0.04)	2.17 (0.03)	2.17 (0.04)	1.851 (0.146)	0.061 (0.980)
Superior part of the precentral S	L	2.08 (0.05)	2.10 (0.05)	2.11 (0.05)	**5.595 (0.002)**	3.815 (0.015)
	R	2.08 (0.05)	2.10 (0.06)	2.12 (0.04)	**5.330 (0.002)**	1.700 (0.178)
Suborbital S	L	1.95 (0.06)	1.96 (0.07)	1.95 (0.07)	0.652 (0.584)	1.105 (0.355)
	R	1.77 (0.12)	1.77 (0.13)	1.80 (0.12)	1.862 (0.144)	0.785 (0.507)
Subparietal S	L	2.09 (0.05)	2.14 (0.05)	2.11 (0.05)	1.485 (0.226)	2.384 (0.079)
	R	2.12 (0.05)	2.16 (0.05)	2.14 (0.05)	1.160 (0.331)	1.878 (0.144)
Inferior temporal S	L	1.96 (0.05)	1.97 (0.03)	1.94 (0.05)	2.123 (0.105)	2.682 (0.056)
	R	1.94 (0.06)	1.95 (0.04)	1.94 (0.05)	0.249 (0.862)	1.304 (0.283)
Superior temporal S	L	2.24 (0.03)	2.27 (0.02)	2.26 (0.02)	2.189 (0.097)	3.180 (0.031)
	R	2.25 (0.03)	2.28 (0.03)	2.27 (0.02)	3.750 (0.015)	2.122 (0.108)
Transverse temporal S	L	1.91 (0.06)	1.93 (0.05)	1.92 (0.04)	1.072 (0.367)	1.847 (0.150)
	R	1.87 (0.06)	1.89 (0.06)	1.89 (0.06)	1.340 (0.268)	0.781 (0.510)

* F (GLM with age and hand lateralization as covariates of no interest; degrees of freedom=3), p threshold determined based on FDR < 0.025. Significant effects that are consistent across both hemispheres are highlighted in **bold**.
